# SeedUSoon: A New Software Program to Improve Seed Stock Management and Plant Line Exchanges between Research Laboratories

**DOI:** 10.3389/fpls.2017.00013

**Published:** 2017-01-20

**Authors:** Céline Charavay, Stéphane Segard, Nathalie Pochon, Laurent Nussaume, Hélène Javot

**Affiliations:** ^1^Institut de Biosciences et Biotechnologies de Grenoble-Laboratoire Biologie à Grande Échelle, Université Grenoble AlpesGrenoble, France; ^2^Institut de Biosciences et Biotechnologies de Grenoble-Laboratoire Biologie à Grande Échelle-Groupe Informatique pour les Scientifiques du Sud Est, Commissariat à l’Energie Atomique et aux Énergies Alternatives (CEA)Grenoble, France; ^3^Laboratoire Biologie à Grande Échelle, Institut National de la Santé et de la Recherche Médicale (INSERM)Grenoble, France; ^4^Laboratoire Biologie Develop Plantes, Institut de Biosciences et Biotechnologies, Commissariat à l’Energie Atomique et aux Énergies Alternatives (CEA)Saint-Paul-lez-Durance, France; ^5^Centre National de la Recherche Scientifique (CNRS) , UMR 7265 Biologie Végétale et Microbiologie EnvironnementalesSaint-Paul-lez-Durance, France; ^6^Aix Marseille Université, BVME UMR 7265Marseille, France

**Keywords:** software, database, plant, seed, genetics, genealogy, MTA

## Abstract

Plant research is supported by an ever-growing collection of mutant or transgenic lines. In the past, a typical basic research laboratory would focus on only a few plant lines that were carefully isolated from collections of lines containing random mutations. The subsequent technological breakthrough in high-throughput sequencing, combined with novel and highly efficient mutagenesis techniques (including site-directed mutagenesis), has led to a recent exponential growth in plant line collections used by individual researchers. Tracking the generation and genetic properties of these genetic resources is thus becoming increasingly challenging for researchers. Another difficulty for researchers is controlling the use of seeds protected by a Material Transfer Agreement, as often only the original recipient of the seeds is aware of the existence of such documents. This situation can thus lead to difficult legal situations. Simultaneously, various institutions and the general public now demand more information about the use of genetically modified organisms (GMOs). In response, researchers are seeking new database solutions to address the triple challenge of research competition, legal constraints, and institutional/public demands. To help plant biology laboratories organize, describe, store, trace, and distribute their seeds, we have developed the new program SeedUSoon, with simplicity in mind. This software contains data management functions that allow the separate tracking of distinct mutations, even in successive crossings or mutagenesis. SeedUSoon reflects the biotechnological diversity of mutations and transgenes contained in any specific line, and the history of their inheritance. It can facilitate GMO certification procedures by distinguishing mutations on the basis of the presence/absence of a transgene, and by recording the technology used for their generation. Its interface can be customized to match the context and rules of any laboratory. In addition, SeedUSoon includes functions to help the laboratory protect intellectual property, export data, and facilitate seed exchange between laboratories. The SeedUSoon program, which is customizable to match individual practices and preferences, provides a powerful toolkit to plant laboratories searching for innovative approaches in laboratory management.

## Introduction

Basic research in plant biology frequently relies on plants whose genomes have been engineered for distinct purposes. For example, biotechnological applications derived from the machinery of the plant pathogen *Agrobacterium* allows the now routine insertion of T-DNA from specific vectors into the plant genome (Hellens et al., 2000). Inserted sequences can allow the expression of a vast array of constructs of interest (such as RNAi or antisense transcripts, GFP-protein fusions, over-expressed genes, biosensors, reporters, and antibiotic or herbicide resistance cassettes). In addition, the insertion of T-DNA into the genome is used to generate libraries of knock-out (KO) mutants. These insertions occur randomly in the plant genome, even though collections of T-DNA insertional mutants will often be enriched for insertions occurring within transcriptionally active parts of the genome (Ortega et al., 2002; Kim and Gelvin, 2007). Similarly, libraries of insertional mutants have been built on the ability of transposons to replicate and insert randomly into the plant genome (Greco et al., 2001; Wegmuller et al., 2008). In addition to collections of T-DNA or transposon insertional mutants, researchers have access to libraries of plant lines containing point mutations or deletions that randomly affect endogenous gene sequences through EMS treatments or irradiations (Li et al., 2002; Kim et al., 2006; Svistoonoff et al., 2007).

Recently, the panel of available mutations was further expanded by engineering site-specific nucleases derived from CRISPR/Cas9, TALEN, ZFN, and meganucleases (Fauser et al., 2014; Baltes and Voytas, 2015). These techniques now make it possible to generate random or precise mutations within specific gene loci in plants, by performing site-directed mutagenesis. Each mutagenesis often results in the generation of a whole set of mutant alleles for a single targeted sequence. Due to their simplicity, some of these applications are becoming routine methods for synthetic biology applications and basic research purposes. These genetic modifications are not only restricted to DNA, as pentatricopeptide repeat proteins also make it possible to alter RNA (Yagi et al., 2014).

The wide availability of efficient and affordable cloning, mutagenesis and transformation techniques has accelerated the generation of transgenic plants. In parallel, the availability of mutant libraries combined with the development of high-throughput sequencing methods has impressively facilitated the precise genotyping of KO mutants. As a consequence, the size of plant and seed collections has dramatically expanded in the past 10 years for many typical research laboratories. These collections contain lines derived from a large diversity of mutagenesis methods, reflecting the ever-growing power of genetics. Plant lines can also combine several mutations, and today it is common to analyze triple, quadruple or quintuple mutants for different loci that were obtained through combinations of different mutagenesis technologies. A clear understanding of the genetic diversity and biotechnological origin of these seed collections is becoming more and more crucial, as each technique presents different risks of artifacts. For instance, CRISPR/Cas9 mutagenesis presents risks of off-target mutations (Baltes and Voytas, 2015), and EMS-mutagenized collections often contain numerous point mutations in a single plant (Henikoff et al., 2004). This can complicate phenotype studies, giving undesired effects that are unrelated to the targeted gene. In addition, some particular sequences (such as the 35S promoter) are known to trigger progressive T-DNA silencing after each successive generation (Mlotshwa et al., 2010). This underscores the importance of maintaining a clear overview of the progeny of a seed (including amplifications), as well as a record of the history of T-DNA inheritance through crosses with other plant lines, or through secondary mutagenesis of lines already containing a T-DNA insert.

Another critical factor is the maintaining of an accurate record of all stored plant lines to comply with procedures linked to genetically modified organism (GMO) certifications. Although the definition of GMOs itself is a matter of debate, the current European regulation distinguished GMOs based on the techniques used for the biotechnological engineering of plants (Hartung and Schiemann, 2014). It will distinguish between plants that contain recombinant DNA from other organisms (classified as GMOs) and plants that contain only point mutations of their native DNA (considered non-GMOs). Transposons are a specific case, depending on whether or not the sequence of the native transposons has been engineered (Greco et al., 2001; Wegmuller et al., 2008). The ability to distinguish mutations on a biotechnology basis (i.e., the presence or not of T-DNA or transposon transgenes versus point mutations) would be a first step toward the improved tracking of plant lines for GMO certifications.

Seed collections are often the result of combined efforts from several people and different laboratories. Resources can be obtained through seed stock centers or by directly contacting the laboratories that generated them. Although the use of most plant lines is often unrestricted, some lines are protected under a Material Transfer Agreement (MTA) signed between research institutions, defining a strict set of acceptable uses of the seeds. It is important to track the original plants protected by an MTA or under the control of a GMO certification as well as their progeny, through all the series of successive seed amplifications and crosses with other lines. The possible use for all of these related plants is equally constrained within the limits of signed MTAs or GMO certification documents. New tools that facilitate tracking of GMOs and MTAs for scientists would greatly improve the compliance within administrative and legal contexts.

Several affordable or free software programs are presently available to improve plant line management. However, to our knowledge none of them are capable of reflecting the inheritance patterns of individual mutations through the successive rounds of seed amplifications, line crossing or mutagenesis encountered in a typical research laboratory. Indeed, most programs have been designed for managers of plant transformation or greenhouse facilities that use standardized procedures (Scott et al., 2003; Henry et al., 2008; Kohl and Gremmels, 2010; Hanke et al., 2014), or for plant breeding laboratories facing large sets of phenotyping and genotyping data derived from accession sequencing or QTL mapping (Lee et al., 2005; Jung et al., 2011; Milc et al., 2011; Love et al., 2012). Nevertheless, several software programs have been developed to track plant lines in basic research laboratories, such as PlantDB and Phytotracker (Exner et al., 2008; Nieuwland et al., 2012). Although they include such functions as plant, seed and plasmid management modules along with genotyping indications, these programs are not capable of independently tracking individual mutations through successive crossings and seed amplifications. In fact, they can only follow the general relationship between seed batches, tracking all seed batches derived from an individual plant.

Despite the presence of these programs, we could not identify any software designed to specifically track the inheritance of mutations or transgenes within the complex history of seed collections, which would also be capable of reflecting the ever-growing diversity of biotechnological applications for plant mutagenesis and transgenesis. We therefore decided to create a new seed and plant database solution that utilizes strong genetic concepts, including mutation inheritance and independent genotyping of each mutation. At the same time, we wanted to provide a simple and intuitive interface that would respect the habits of individual laboratories and their members.

To answer this need, we have developed the “SeedUSoon” software. Its intuitive and flexible user interface permits the tracking of plant lines along with plants and seed batches, and it includes a graphical representation of the genetic link between related plant lines arising from crosses or secondary mutagenesis. Mutations inherited from parental lines can easily be identified using our software, and transgenic (GMO)/non-transgenic (non-GMO) types of mutations can be color-coded for fast visual identification.

The program can be easily customized to the needs of each laboratory through an administrative module, for use with different plant models or mutagenesis techniques, for instance. Users can also decide whether they enter each seed generation, or only important seed batches. Other functions include the uploading of genotyping protocols (for instance PCR primers and programs used to identify a mutation), articles, genotyping, and the phenotyping results of individual samples, microscopy images, etc. We have also developed export/import functions to facilitate seed exchange between laboratories, and MTA tracking functions for improved intellectual property management practices. Altogether, the SeedUSoon software is an attractive and free solution for plant laboratories facing the challenge of keeping accurate seed collection records.

## Materials and Methods

### Implementation

We developed the SeedUSoon user interface (version 1.1.0) using the platform-independent Java programming language^1^. This choice allows the software to operate on any system running Java 1.8 or higher. It has been tested with the Windows XP, Windows 7, Windows 8, and Mac OS X (up to Mavericks) operating systems.

The SeedUSoon software needs to connect to a database (client/server architecture) that can be present on the same computer, or preferably on a server for multiple user access. The computer hosting the database must run MySQL (version 5.5.35 or higher). Extended computer knowledge is only necessary for the database installation.

### Software and Start-Up Database Availability

SeedUSoon is distributed under a proprietary license, and is free exclusively for academic purposes (i.e., non-profit institutions). Non-academics interested in the program cannot access the software and must contact us directly.

Academics can sign the proprietary license agreement through the project website^2^. Once completed, this provides access to the download page for the SeedUSoon software, a start-up database, and the installation procedures.

An example of exported data, a template form to load customized laboratory information, demonstration movies, FAQs and access to updated versions of the software will be posted on the project website. Specific questions can be directed to the project leaders by using the dedicated email address SeedUSoon@cea.fr.

## Results

### SeedUSoon Concepts

#### “Line” Concept

**FIGURE 1:**
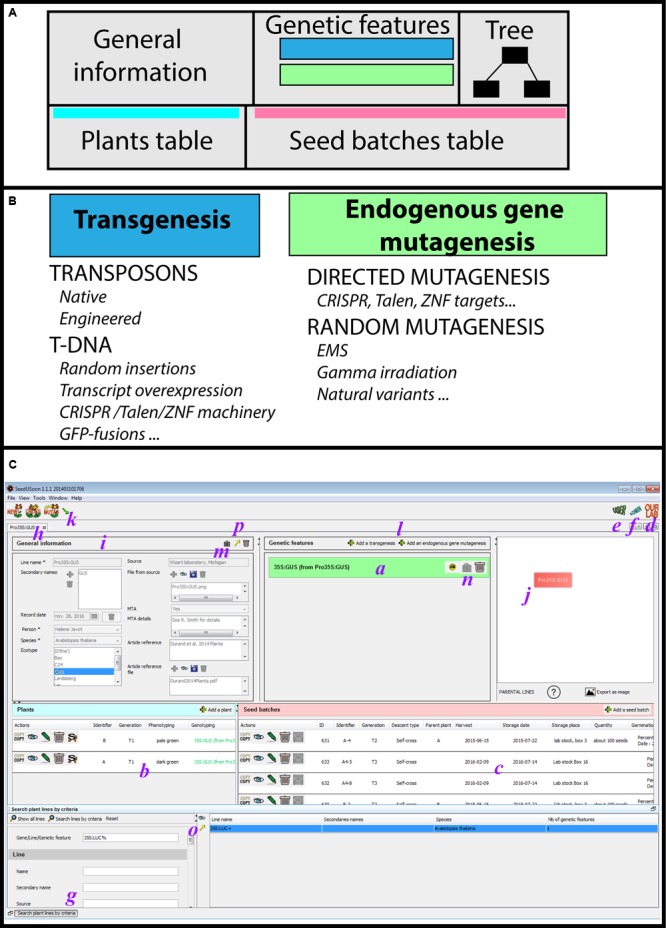
**User mode interface. (A)** General organization of a plant line datasheet. **(B**) The two categories of genetic features (transgenesis/endogenous gene mutagenesis) with examples of corresponding applications. **(C)** Detailed user mode organization; ***a***: genetic features table, ***b***: plant table, ***c***: seed batches table, ***d***: access to the customized laboratory guidelines, ***e***: access to the user mode, ***f***: access to the administrative mode, ***g***: search engine, ***h***: plant line datasheet, ***i***: general information, ***j***: genealogy tree, ***k***: access to plant line generation wizards (new, crossing, mutagenesis, and import), ***l***: addition of new genetic features, ***m,n***: lock buttons, ***o,p***: export buttons.

SeedUSoon is designed around the core concept of “plant lines,” whose definition is very similar to the one used by many plant science researchers when referring to the series of successive generations derived from particular plants. A “Plant line” is defined by a set of unique traits (mutations or transgenes, named “Genetic features” in SeedUSoon; **Figures 1A,B**) in a biological context (species and ecotype). All plants and seeds arising from selfcrosses or backcrosses are still considered part of the same “Plant line,” so that under a single “Plant line” entry, the user can record as many seeds or plants as desired. The precise genotyping (such as the heterozygous/homozygous state of each mutation, or the segregation pattern of seed batches) can be recorded for each individual plant or seed batch entry.

However, if crosses are performed with plants carrying other types or mutations or with plants from another ecotype, the genetic properties of the resulting line will significantly differ from the original plant. For this reason, the progeny of these types of crossings must be entered as a new “Plant line” entry. Similarly, if a previously mutagenized plant line is subjected to a secondary mutagenesis or transgenesis, the resulting progeny will constitute a new “Plant line.”

A “Plant line” datasheet is organized in five different parts (**Figure 1A**). The line name and general information associated with this line are displayed in the upper left area, including the plant species and ecotype, its origin and the existence of any MTA protecting the material (“General information” fields, **Figures 1A,C**, Supplementary Figure 1). In the center of the screen, a table presents blue or green boxes containing the names of all individual mutations or transgenes (i.e., “Genetic features”; **Figures 1A–C**) along with their origin (in which “from…” indicates the original plant line containing this mutation). The content of each box can be expanded in order to access the individual properties of each “Genetic feature” (“Genetic features fields,” Supplementary Figure 1). If no green or blue box appears inside this table, the genotype of the corresponding plant line is considered to be WT. At the upper right corner, the parental lineage and the mutagenesis/genetic history of the opened plant line datasheet is represented by a tree.

Finally, two tables for plants (**Figures 1A,C**, left) and seed batches (**Figures 1A,C**, right) are located in the lower part of the datasheet. Generations of plants and seeds can be recorded at any time, including the skipping of generations, which allows users to avoid a strict “generation workflow.” The user enters the generation stage and can record for each plant the precise genotyping of each “Genetic feature” listed in the upper table (or the segregation profile for each seed batch). Consequently, by using a “Plant line” as the entry point, the user can access all recorded generations of seeds or plants that share the same overall genetic background.

By organizing the datasheet into five parts (general information, genetic features, tree, the plants table, and the seed batches table), users can focus on the core properties of each line (genetic context and history, ecotype, etc.) before searching through all available seeds or plants corresponding to these criteria. The software also allows users to decide whether they want to track all successive generations of a plant line, or to only record a subset of particularly valuable seed batches.

During the development of our software, the ability to compare the behavior and properties of successive seed generations in a single table was a recurring request from researchers that we spoke with. One reason for this is that epigenetic phenomena can affect the behavior of descendants of seemingly identical plants, in particular through DNA methylation (Mlotshwa et al., 2010; Diez et al., 2014). A table comparing the properties of distinct generations can thus be instrumental in identifying the appearance or loss of specific phenotypes, or the progressive silencing of T-DNA expression. Similarly, the lack of a link between phenotype and desired mutations might suggest the presence of an unknown off-target mutation (frequent with CRISPR/Cas9 or EMS mutagenesis).

When performing T-DNA transformation for specific purposes (such as RNAi silencing or expression of GFP-protein fusions), it is convenient to visualize all available independent transformants at once (along with their descendants). In this case, a software user often prefers to record all independent transformants within a single “Plant line” rather than as separate “plant lines.” Properly speaking, the independent transformants do not share the exact same genetic properties. The T-DNA insertion sites are variable in these lines, and have a putative impact on the properties of the resulting plants (Kohli et al., 2006). Nevertheless, comparing all independent transformants within a single plant line can be advantageous for tracking the outcome of a T-DNA transformation; this approach can also quickly identify undesirable effects, such as construct silencing, patchy expression, etc. With SeedUSoon, laboratories can decide if they want to record independent plant line datasheets for each insertion or use a single datasheet for all independent transgene insertion events, since T-DNA insertion sites can be defined in two locations within the datasheet: either in the transgene sequence section in the “Genetic feature” table (**Figure 1A**), or for each of the individual plants recorded in the lower table (i.e., different insertion sites can be recorded in the plant table specifically for each plant entry; **Figures 1A,C-a,b**). Finally, seed batches can be linked to these individual plants.

#### Two Categories of “Genetic Features”

Mutations or transgenes present in the genome of a “Plant line” are recorded as unique “Genetic Features,” and listed in the corresponding table of the plant line datasheet (**Figure 1A**). There are two distinct feature categories, “Transgenesis” and “Endogenous gene mutagenesis,” with the latter one corresponding to point mutations, nucleic deletions and insertions affecting an endogenous genomic sequence that does not involve the insertion of a transgene (T-DNA or transposon).

The “Transgenesis” feature must be selected for T-DNA or transposon mutagenesis (Clough and Bent, 1998; Greco et al., 2001; Gomez et al., 2009), and corresponding mutations will appear in green in the features table (**Figure 1B**). “Endogenous gene mutagenesis” corresponds to mutations in endogenous genes with no transgene insertion, such as EMS, gamma irradiation, or natural variants (Kim et al., 2006; Svistoonoff et al., 2007; Fauser et al., 2014). These will appear in blue in the features table (**Figure 1B**). In the case of CRISPR, TALEN, or ZFN mutagenesis, the plant line will contain both a “Transgenesis” box in green (i.e., the T-DNA containing the mutagenic machinery) and an “Endogenous gene mutagenesis” box in blue [i.e., the targeted endogenous gene loci (Fauser et al., 2014)].

The blue/green color code allows the user to quickly recognize the “Transgenesis” from “Endogenous gene mutagenesis” features. Plants potentially containing transgenes (i.e., GMOs) can thus be immediately distinguished from all other mutation categories (**Figure 1A**).

Each feature category will call for a specific set of information fields that are ready to be completed by the user (Supplementary Figure 1). In particular, a single sequence can be recorded for “Endogenous mutagenesis” (the mutated genomic locus), whereas a “Transgenesis” feature can record the transgene sequence (in the “Genetic features” properties) as well as several independent genomic insertion sites (in the plant table entries; **Figure 1A**).

#### Easy Customization of SeedUSoon: Adaptation to the Laboratory Context

##### Personalized “lab” guidelines

SeedUSoon contains a customizable document that will provide laboratory members with specific guidelines and rules decided within their own laboratory. An “Our lab” icon is always visible on the SeedUSoon main page and provides access to this document (**Figure 1C-d**). This document specifies how to name lines and successive generations, and describes which file formats are acceptable for upload into the database. In addition, it provides details on the organization of the laboratory’s common seed stock, how to store seeds, and protocols for seed selection, transformation, etc.

A window asking the user to upload the manual will appear following the first activation of the “Our lab” icon. After this initial upload, the document will automatically open whenever any user clicks on “Our lab.” Newer versions of the manual can then be uploaded by following the path: Tools tab/Options/Labo/User manual.

A document containing an example of laboratory guidelines is provided for use as a template (see Supplementary Data Sheet 1 and the project website).

##### Customization of the user module

Parameters and methods susceptible to change between laboratories are presented in scroll-down menus when in the user mode. These menu options are customizable, but can only be edited by the database administrator in the administrative mode (see the corresponding section for details). This allows the software options to closely match the habits and protocols of each laboratory, while preserving a certain consistency.

Through these scroll-down menus, the user will have access to a specific selection of laboratory member’s names, plant species, ecotypes, strains, plant resistances, and mutation methods in use in the laboratory. New entries or modifications to the scroll-down menu options can be made at any time during the database lifetime, and corresponding plant lines will be updated accordingly.

### User Mode

After starting the software, the user interface can be accessed from the home page by clicking on “User” (**Figure 1C-e**).

#### Built-In Pop-Ups

**FIGURE 2:**
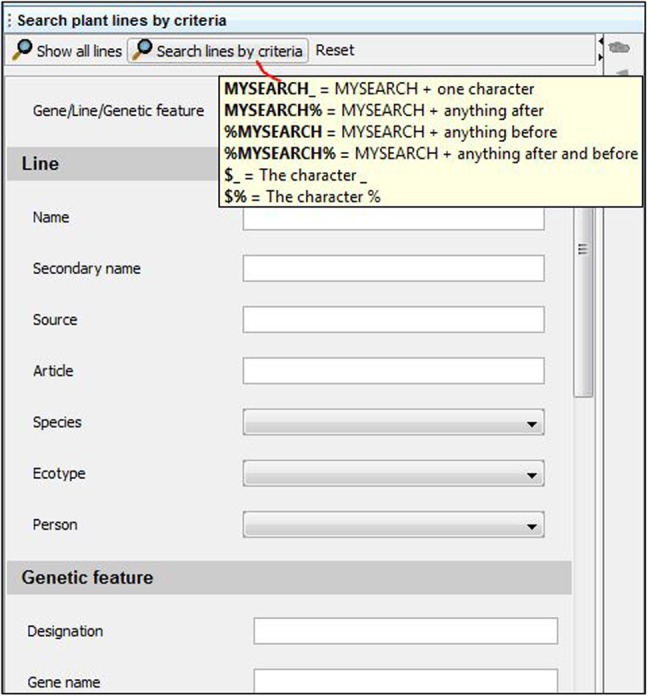
**Search engine interface.** Key words can be entered in a generic field (“Gene/Line/Genetic feature” field) or in specific sub-categories to narrow the search. A “%” symbol can be included to replace any number of characters in the query. Including a “_” indicates the presence of a single missing unknown character in the query. Adding a “$” before “_” or “%” in the query field will allow the user to search for items containing the characters “_” or “%,” bypassing the use of these symbols as replacements of any characters in the query.

Scrolling the mouse pointer over most fields or icons activates pop-ups that provide more information to the user about the purpose of these functions (**Figure 2**). In some cases, pop-ups will recommend reading the “Our lab” document mentioned in the previous section, to ensure that users will follow the specific rules that have been decided for their laboratory.

#### Searching for Available Plant Lines or Seed Batches

A search engine is located at the bottom of the user interface (**Figures 1C-g** and **2**), which can provide access to all “Plant lines” present in the database (by clicking on “Show all lines”), or only a subset of lines when “Search lines by criteria” is selected (**Figure 2**). The first field (“Gene, Line, Genetic feature”) can be used to search a keyword throughout all plant line names, gene names and genetic features recorded in the database. Alternatively, users can select more restrictive query criteria by completing the fields specifically associated with the 4 individual sub-parts of a plant line datasheet. These fields can be among the general properties of the line, genetic feature properties, and plant or seed batch properties (including seed batch name or ID, person involved, etc.; **Figures 1A** and **2**; Supplementary Figure 1).

When there is some uncertainty regarding the exact spelling of a query, a “%” symbol can be included at the beginning or end of the word (**Figure 2**). This will identify any lines containing the searched criteria, including any number of characters appearing before or after the searched word (i.e., “%” = any number of characters).

Clicking on the name of a plant line in the search engine result table will open the corresponding datasheet (**Figure 1C-h**).

#### Creating a Plant Line

There are four possible ways to create a new line in SeedUSoon: through the generation of a “New record,” crossing, secondary mutagenesis or by import. A button corresponding to each mode is located at the upper left corner of the user interface (**Figure 1C-k**).

##### New record in the database

**FIGURE 3:**
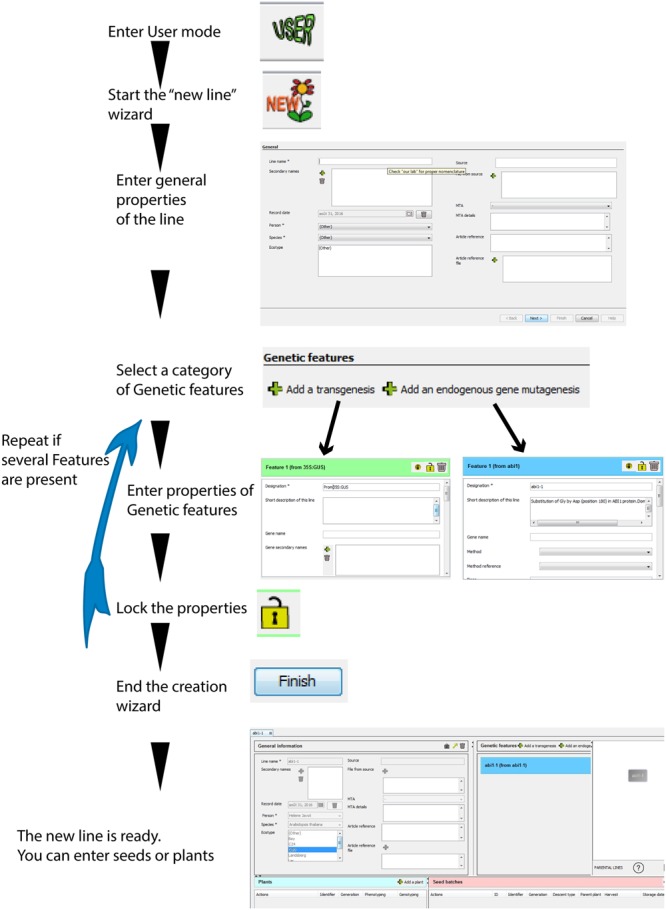
**Principle steps in the new plant line wizard.** A new plant line can be recorded *de novo* following these successive steps. One or several “Genetic features” can be included during the creation process. The resulting “tree” shows that this line does not depend on any parental lines present in the database (no parental lines indicated in the tree). The icons at the bottom provide access to the number of descendants from the plant line, and to an export function of the graphical representation.

The user can create a “Plant line” record *de novo*, by entering any available information in the empty fields of the new database entry (**Figure 3**). Most fields are optional and can be completed later (mandatory and facultative fields are listed in Supplementary Figure 1) to avoid any wrong assumptions when recording data (arising from erroneous “guess work”). Data can easily be saved, completed or modified at any time by clicking on the lock buttons (**Figures 1C-m,n** and **3**).

When starting the “new” plant line wizard, the user will only be required to complete three mandatory fields: the line name, the person associated with this datasheet, and the plant species. No other additional information is needed in the case of a WT plant.

If the plant line contains one or several mutations (or transgenesis), the user can click on “Add a transgenesis” or “Add an endogenous gene mutagenesis.” This will select the correct category of “Genetic Features” to appear in the “Genetic features” table (**Figures 1B,C-a**) with new empty fields related to these specific mutations (such as gene, mutagenesis method, transgene or mutation sequence, attached sequence files, selectable marker in plants, etc. see Supplementary Figure 1). Only the “designation” (i.e., a mutation name) is required for each genetic feature, which allows their quick recognition in the “Genetic features” table (for example: Pro35S:GUS). In the “Genotyping protocol” field, the user can type or upload a standard genotyping method for this particular feature (including PCR primers, PCR programs, a picture of a typical gel, etc.).

The user should add as many “Genetic features” as the line contains mutations or transgenesis. The different mutations can all be recorded during the “New” plant line wizard, or can be completed after a plant line is created (by clicking on the buttons located over the genetic features table; **Figure 3**).

##### Crossing two previously recorded plant lines

SeedUSoon is capable of predicting the genetic configuration of plants resulting from the crossing of two plant lines that are already present in the database. The software will import all important properties from the parental lines and create a new plant line that combines this data (mix of ecotypes, set of combined genetic features, etc.). Only intra-species crosses are permitted by the software.

**FIGURE 4:**
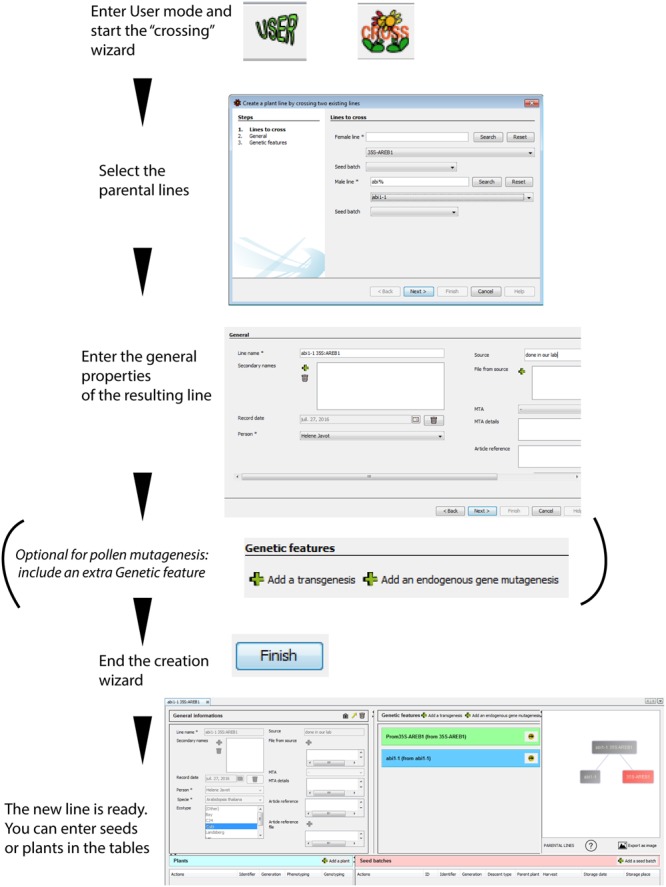
**Simulated crossing of two lines using the “cross” wizard.** A new plant line can be created using the simulated crossing between two parental lines present in the database. This line will inherit all genetic features from the parents. It is possible to add an extra genetic feature during the crossing process for the specific case of pollen mutagenesis, where crossings are combined with additional mutagenesis. The resulting “genealogical tree” reflects the relationship between the parental and resulting plant lines. A plant line protected by an Material Transfer Agreement (MTA) is indicated in red. The icons at the bottom provide access to the number of descendants from the plant line, and to an export function of the graphical representation.

After starting the “Cross” wizard (**Figure 4**), the user will only need to specify which parents were used as male/female; optionally, the seed batches used for the crossing can be included. This will generate a new datasheet, to which the user can allocate corresponding seeds or plants (the user can also specify whether the mutations are homozygous or heterozygous). To avoid mistakes, the user cannot modify the inherited properties, as these come from the parental lines. Any modifications should therefore be entered in the parental line, and all descendants will be updated accordingly. All non-inherited fields can be edited.

##### Secondary mutagenesis of a recorded plant line

**FIGURE 5:**
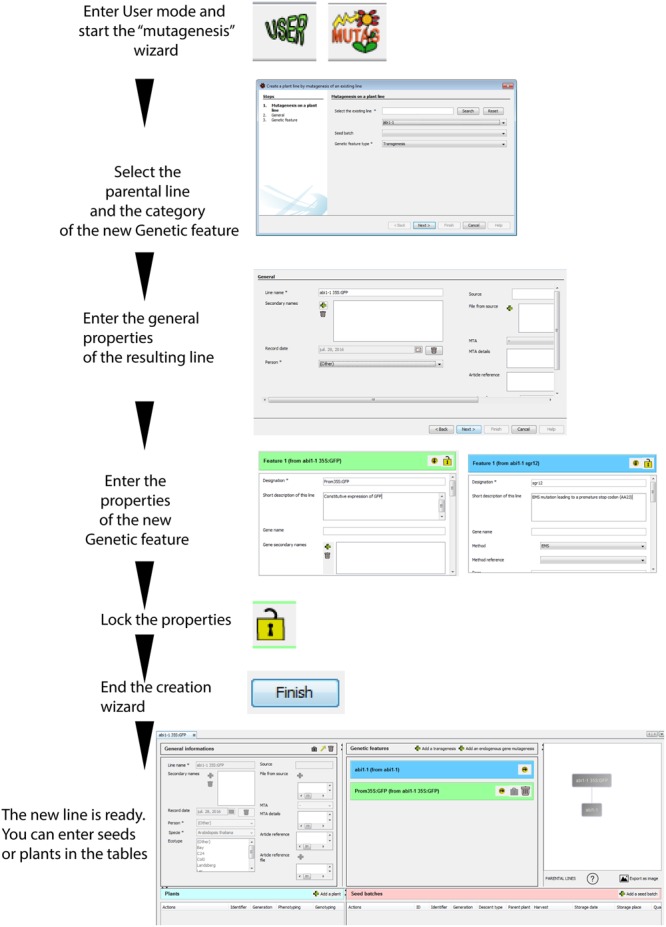
**Creation of a line using the “MUTAG” wizard in order to apply a secondary mutagenesis to an existing plant line.** A new plant line can be created by secondary mutagenesis (addition of a genetic feature) to a parental line already present in the database. The new line will inherit all genetic features from this parental line, and combine it to the new genetic feature. The resulting “genealogical tree” reflects the relationship between the parental and resulting plant lines. The icons at the bottom provide access to the number of descendants from the plant line, and to an export function of the graphical representation.

The “MUTAG” wizard can be used if a new mutagenesis has been applied to a plant line previously recorded in the database (**Figure 5**). As with the “Cross” wizard, “MUTAG” will import all the genetic properties from the mother line into the new line (species, ecotype, and genetic features). The user will only need to complete the fields corresponding to the new “Genetic feature” selected for the secondary mutagenesis (**Figure 5**).

Similarly to the “Cross” wizard, the data inherited from the parental line cannot be edited in the datasheet of the resulting line.

##### Importing/exporting a line

**FIGURE 6:**
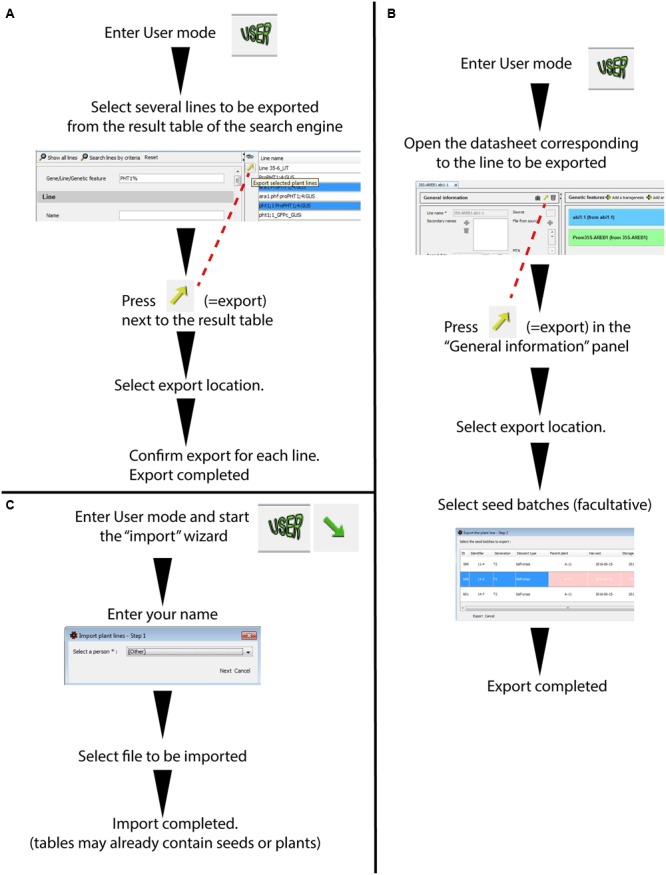
**Wizards for plant line export/import between SeedUSoon databases. (A)** Batch export of plant lines through the result of the search engine (without seed batches attached). **(B)** Single export of a plant line with attached seed batches (from a plant line datasheet). **(C)** Plant line import from a .json file into a SeedUSoon database.

It is possible to export a database entry from a “Plant line” (with or without the corresponding seed batches) into a single file that can easily be sent to collaborators (**Figure 6**). This file is generated using an exchange format (.json). Although this format can only be partially read in a text processor such as WordPad, it permits a very complete data exchange between two SeedUSoon databases, including all attached files (plasmid sequence, phenotyping results, etc.).

The export can be performed either from the Research result table (**Figure 6A**) or directly from the opened plant line datasheets (**Figure 6B**). The simultaneous export of several lines is only possible through the Research result table, however, no seed batches can be exported along with the line information in this case. When exporting directly from the plant line datasheet, it is possible to assemble the information from one or several seed batches (with the exception of sensitive/personal information; see Supplementary Figure 1). Any plant (from the plant table) that is linked to a seed batch (identified as its mother plant) will be exported as well.

Reciprocally, a plant line can be created by importing data from other databases. To import data, the user must click on the “Import arrow” at the upper left corner (**Figures 1C-k** and **6C**), specify a name associated with this new database entry, and select the .json file. If the import contains options that are not available in the database scroll-down menus, a pop-up window will warn the user that the administrator must create the corresponding choices via the administration mode. Alternatively, the missing entry can be edited directly by opening the .json file in a text processor software; this can also serve as a temporary solution if the administrator is not present. For instance, a missing ecotype can be temporarily changed to “(Other)”; this option is included by default in the scroll-down menu.

When lines are imported, all links to parental lines are severed. However, the imported line will contain the proper list of genetic features. The graphical representation of the genealogical tree (refer to the section below) will not be lost: it will be exported as an image, and will be uploaded as a “File from source” in the “General information” panel (**Figure 1C-i**).

Most of the entries related to general information, genetic features and seed batches will be imported (**Figures 1A,C-i** and Supplementary Figure 1). However, any sensitive/personal information (such as any personal name, notebook information, storage place, or MTA details) will not be included in the export format file.

##### Graphical representation of the genealogy of a line

On the right part of any “Plant line” datasheet is displayed a graphical representation of the history (“genealogy” or “tree”) of this plant line (**Figure 1A**) in relation to other plant lines. The purpose of this is not to track successive generations of a single plant line, but rather to represent the links toward the parental “Plant lines” and visualize when new “Genetic features” were brought into the genome of the plants.

A plant line created *de novo* (using the “New” plant line wizard) will be represented by a simple rectangle containing its name (**Figure 3**). If a line is crossed to another one (through the “Cross” wizard), or if it was recorded for a secondary mutagenesis (using the “MUTAG” wizard), both the parental lines and the resulting line will appear in the graphics (**Figures 4** and **5**). The graphical representation will reflect the complete origin of a plant line, even for plant lines resulting from several rounds of successive crossings and mutagenesis.

Although this tree does not directly track the individual mutations, it is easy to infer from the adjacent “Genetic features” table whether any mutations or transgenes are inherited from a parental line. First, inherited genetic features cannot be edited (no “lock” or “trash” icons appear in their corresponding boxes). Second, the boxes contain the name of the plant line from which the corresponding genetic feature (indicated by the name of the feature followed by “from…”) originated. The color code of the boxes (green/blue) permits the fast identification of the category of the genetic feature (transgenic vs. non-transgenic).

The parental line can be directly accessed by clicking on its name in the graphics. In addition, scrolling the mouse pointer over the line connecting two lines displays the seed batches used for their generation in a pop-up (if previously recorded).

The graphics only represent the parental lines of the line of interest. However, placing the mouse pointer over the question mark located beneath the graphic will display a pop-up indicating the number of descendants derived from this specific line (**Figure 1C**).

The parental lines are located at the bottom of the “genealogy” tree in this software version. The tree orientation can be inverted or modified by dragging its individual components inside the graphics window with the mouse.

Graphics can be exported as an image (in .png format) by clicking on “Export as image” underneath the tree (**Figure 1C**), for inclusion in notebooks or PowerPoint presentations. Furthermore, when exporting a line using the SeedUSoon export/import format, the graphical representation of the genealogical tree will be included as an image in the “File from source” field, in the “General information” panel (**Figure 1A**).

#### Recording Plants and Seeds

**FIGURE 7:**
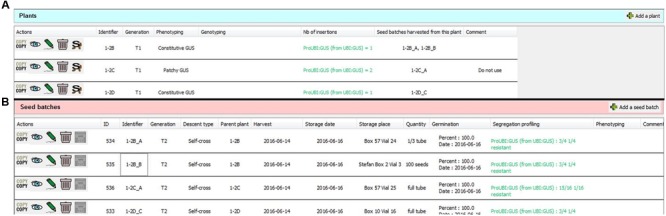
**Two tables for recording biological material corresponding to a plant line.** Samples are separated into two tables: plants **(A)** and seed batches **(B)**. In the example shown here, the researcher decided to record different T1 plants derived from a T-DNA transformation within a single plant line datasheet. The number of insertion sites and location/sequences of independent insertions can be stored separately for each plant. Only the major fields are shown in the columns; detailed information and attached files can be accessed by clicking on the eye icon. It is possible to link the mother plants to their seed batches. The “Copy” button permits fast duplication of plant or seed batch data.

Following the creation of a “Plant line,” it is possible to record the corresponding seed batches or individual plants in two dedicated tables (**Figures 1A,C-b,c** and **7A,B**). These tables can contain any generation of seed batches or plants sharing the same ecotype and set of mutations (i.e., “Genetic features”), including descendants of self-crossed or back-crossed plants. For each plant or seed batch entry, the user can specify their specific genotype or segregation profile (heterozygous/homozygous, single/multiple transgene insertions, resistance or mutation segregation ratio).

The “Plant” and “Seed batch” wizards can be activated by clicking on “Add a plant” or “Add a seed batch” located at the right corner above the plant and seed tables, respectively (**Figures 7A,B**). The only mandatory field here is the personal plant or seed batch identifier. All other information (generation, phenotyping, genotyping, harvest date, etc.) is optional (see Supplementary Figure 1 for the list of available fields) and can be completed later. Generation stages are entered by the user in the corresponding field, according to the recommendations of the customizable “Laboratory guidelines.” These guidelines can request the use of classical terms, such as T1, T2, F1, F3, as well as other terms such as “unknown” or “Tx” when receiving seeds from another laboratory, for example (see Supplementary Data Sheet 1). Although there is no requirement to track all successive generations, it is possible to associate specific plants with their progeny using the “S” (seed batch) function available for each plant table entry (**Figure 7A**). Reciprocally, mother plants from seed batches can be indicated when using the “Seed batch” wizard.

SeedUSoon will assign a unique ID number to each seed batch (in addition to the identifier entered by the user; **Figure 7B**, “ID” column). If a seed batch is deleted (for instance if no seeds are left), this ID number can never be reallocated to any other seed batch. Similarly, even if two seed batches possess the same personal identifier, they will have two unique ID numbers. This software-generated unique ID number therefore provides an easy and secure way to unambiguously distinguish seed batches. This feature can also be used to improve seed stock organization, if included in the label present on the seed stock tubes. SeedUSoon users can simply enter this ID number in the software search engine, and, with this information alone, directly access the corresponding seed batch and plant line information.

An additional field can only be activated for plants resulting from transgenesis, to permit the recording of the location and sequences of one of several insertion sites in individual plants. This facilitates the work of users who prefer to record a series of plants with independent T-DNA or transposon locations within the same table of a unique “Plant line,” rather than in separate “Plant lines” (an example of this application can be seen in **Figure 7B**).

A “Copy” button is located at the left of each table entry to accelerate the recording of similar seed batches or plants (**Figures 7A,B**). Its activation will open a wizard, and the user will only need to validate or edit the duplicated information. For seed batches, the software will allocate a unique ID number to the new entry.

#### Recording Phenotypical Data and Experimental Results

Since phenotypical data are often influenced by plant or seed batches, phenotypical results can be individually recorded for each entry in the “Plant” and/or “Seed batch” tables of SeedUSoon (**Figure 1C-b,c**, Supplementary Figure 1). The user can type a short description of the phenotype in the corresponding wizard field (this text will appear in the table; see the plant example in **Figure 1C-b**). The user can also upload files describing detailed phenotyping results in the same section. Scrolling the mouse over the “phenotyping” section of the table will reveal the presence of the uploaded file.

Results of tissue-expression patterns (such as from GFP-fusion or reporter gene studies) can also be uploaded in this “phenotyping” section of the “Plant” and “Seed batch” tables.

Germination assays, genotyping and sequencing results can also be recorded or uploaded within individual seed or plant batches. The reference number and pages of the laboratory notebook can be indicated for each result section (phenotyping, germination assay, genotyping, etc.), along with the name of the person who conducted the experiment.

#### MTA Tracking

SeedUSoon includes a function to help protect the intellectual property of laboratories, especially related to MTA tracking. An MTA field is included in the plant lines “General information” panel (**Figure 1C-i**). The “MTA details” field allows users to record the recipient of the MTA, its location, or any particular recommendation. When exporting a line, the information regarding the presence of an MTA protecting the line is preserved. However, the “MTA details” field is left empty for confidential reasons.

SeedUSoon’s graphical “tree” representations of plant lines allow users to immediately identify a protected material. Any plant line protected by an MTA will be indicated in red (**Figure 4**), so that tracking its descendants will be straightforward, even long after obtaining and using the original seeds.

### Administrative Mode

The “Admin” icon (**Figure 1C-f**) provides access to the administrative mode (only for users with administrative rights) to be able to customize the user interface, create SeedUSoon user accounts, and specify their rights. All customization choices recorded from the administrative mode (on a single computer) will be effective for all computers that connect to the same database.

**FIGURE 8:**
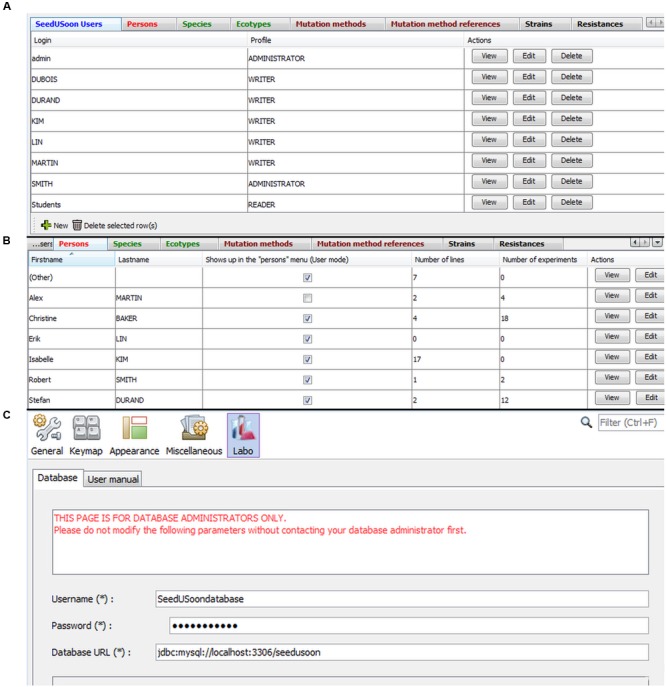
**Administrative mode and database configuration. (A)** The administrative interface is organized in eight tabs: SeedUSoon users (selected here), Persons, species, Ecotypes, Mutation methods, Method references, Strains and Resistances. **(B)** Persons tab, displaying how many lines or experiments are associated with specific names corresponding to laboratory members. **(C)** Database configuration panel.

The administrative mode is a very simple interface organized in eight tabs, each giving access to a table with editable content (**Figure 8A**). A wizard for generating new entries can be activated by clicking on “New” at the bottom of each table. Existing entries can be separately edited or deleted using the buttons at the right of each table.

#### Defining User’s Rights

In the first tab (“SeedUSoon Users”), the administrator can create login accounts and define distinct levels of user’s rights (**Figure 8A**). SeedUSoon users will either be allowed to enter and modify data (“Writer” level), or will only be able to access the data without modifying them (“Reader” level). The final user level (“Administrator”) gives additional access to the administrative mode.

#### Scroll-Down Menu Customization

All tabs (aside from the one used to define “SeedUSoon users”) are dedicated to the customization of the user’s module (**Figure 8B**). This allows the administrator to specify the options available in the scroll-down menus presented to the users. The software comes with a set of pre-recorded options for each tab, which can easily be edited by the database manager. For each tab entry, the software will automatically verify and count lines, features or experiments containing the corresponding scroll-down menu option. This will help the manager visualize the relevance of certain fields, in order to only delete unused menu options.

##### Persons

The “Persons” tab (**Figure 8B**) corresponds to current or past laboratory members who contributed to the generation or the analysis of any plant line recorded in the database. This category should not be confused with the previously mentioned “SeedUSoon users” category. If a “Person” leaves the laboratory and no new entries will be generated under this name, it is possible to deactivate (hide) the name from the scroll-down menus when generating new plant line entries. This limits the length of scroll-down menus in laboratories with a high turnover of members. To do this, the administrator must uncheck the box “Shows up in the “Persons” Menu (User mode)” in the central column of the “Persons” table (**Figure 8B**). Previous entries containing a reference to this person will still display the name.

##### Species and ecotypes

Laboratories can enter their plant models and favorite ecotypes in the “Species” and “Ecotypes” tabs. In the scroll-down menus of the user mode, ecotypes will be specific to each species. For this reason, in the administrative mode, new species must be recorded before registering ecotypes, so that the correct species can be linked to the new ecotype when creating new entries in the ecotype tab.

##### Mutation methods

The “Mutation methods” tab contains the common short terms used to refer to standard mutagenesis techniques used in the laboratory (such as: “T-DNA,” “CRISPR,” “EMS,” etc.). When specifying a new “Mutation method,” the administrator must first define its “Genetic features” category (i.e., “Transgenesis” or “Endogenous gene mutagenesis”; **Figure 1B** and refer to the dedicated section).

The “Mutation method references” tab can be used to record a precise reference from the literature or a precise protocol registered in the laboratory.

Finally, *Agrobacterium* strains and resistances originated by T-DNA or transposon insertions in the plant genome can respectively be recorded in the “Strains” and “Resistances” tabs.

### Database Configuration

Database connection parameters must be entered at the first startup of the software (**Figure 8C**). The software will then restart to allow users (or administrators) to enter their login and password to access the user (or administrative) mode after this initial configuration.

If some users need to connect to a different database, these connection parameters can be modified by following the path: Tools tab/Options/Labo/Database.

## Conclusion and Future Developments

SeedUSoon is a new plant line database software, built upon a strong genetic foundation. The software’s ability to track the history of mutations or transgene inheritance, in addition to the possibility to record related seed batches, provides the user with a more clear and organized view of the genomic context of their biological material. SeedUSoon contains novel functions related to MTA tracking and easily distinguishes between GMO and non-GMO plant lines, to facilitate administrative and legal compliance. Exporting data between databases is also greatly simplified by the import/export functions.

Our intention when we started to design SeedUSoon was to improve the management of our own laboratory plant lines and seed collections. Nevertheless, from the start, the software was also meant to be able to adjust to the context and habits of any other plant laboratories conducting basic research. We achieved this goal by developing a customizable user’s module, and by integrating choices for field entries that are respectful of individual user habits. To help managers or PIs standardize entries in their own database or seed collection, a customizable “Laboratory guidelines” document is easily accessible from the software.

Several additional functions were requested during the development of SeedUSoon. The current version of the software was designed in order to implement most of these requests in the future. For instance, the possibility to connect to different SeedUSoon databases using a simple Login/Logout could be advantageous to access distinct databases dedicated to specific projects. We also took into account future functions that can print labels (with customizable content, including unique ID numbers and plant line names), or export data in a diversity of formats (to generate files necessary for GMO certification, for instance). In collaboration with our Intellectual Property department, we considered the possibility of generating MTAs prefilled with plant line information, which would only require the addition of the recipient identification and the approval signatures. This feature would greatly facilitate and stimulate this procedure, since the signing of MTAs when sending seeds to other research groups is hard to implement in many laboratories.

User feedback (through the project website and the dedicated email address) will be important in helping us decide on the strategy for future SeedUSoon developments. Similarly, the design of the current version was improved by the feedback from users of previous versions of SeedUSoon. In the current configuration, this software has already helped laboratories organize hundreds of plant lines, from their generation to the organization of seed collections. Several plant biology laboratories from our research organization have implemented SeedUSoon in recent years, and it is now available for broader distribution (under the protection of a proprietary license agreement).

The design of this software is intended to help others optimize the tracking of their biological material. Ultimately, SeedUSoon will contribute to a facilitated and improved exchange of information to accompany seed exchange between laboratories.

## Author Contributions

HJ, CC, LN, and NP designed the functional aspect of the software. CC and SS programmed the software. HJ drafted the manuscript. All authors tested the software, and have read and approved the final manuscript.

## Conflict of Interest Statement

The authors declare that the research was conducted in the absence of any commercial or financial relationships that could be construed as a potential conflict of interest.

## Footnotes

1 https://www.java.com

2 http://biam.cea.fr/drf/biam/Pages/laboratoires/lbdp/SeedUSoon.aspx
