# Supplementary material for: SeedUSoon: A New Software Program to Improve Seed Stock Management and Plant Line Exchanges between Research Laboratories
Source: Front Plant Sci. 2017 Jan 20;8:13. doi: 10.3389/fpls.2017.00013 (PMC5247430; doi:10.3389/fpls.2017.00013)
Supplement: Supplementary file 1 [file Data_Sheet_1.DOCX]

*The following quidelines are only provided as an example. Feel free to edit/enrich/simplify this document.*

OUR LAB GUIDELINES FOR SEED HANDLING/NAMING, ETC.

**Prepared by:** H. Javot (original SeedUSoon template)

**Laboratory name:** SeedUSoon team **Date:** Nov. 2016

NAMING YOUR LINES

In the field **“Line name”,** enter a name which respects international conventions. This is the name that should be used for any publication.

PLEASE NOTE: YOU CANNOT USE ITALICS IN THE SOFTWARE (unfortunately!)

If the precise mutation of the line has not yet been characterized, **enter a temporary name and specify this by adding “temp”** at the end (for instance: revert4 temp).

In the field **“secondary names”,** you can enter all the names in use in the laboratory for this line

**Each plant species follows different rules regarding nomenclature.** A good place to start is the **“Plant Cell” website,** which provides links to the nomenclatures of the major plant models.

[**http://www.plantcell.org/site/misc/ifora.xhtml#Nomenclature%20and%20Terminology**](http://www.plantcell.org/site/misc/ifora.xhtml#Nomenclature%20and%20Terminology)

**Gene fusions and constructs.**

Promoters should be written Pro (e.g., Pro35S) and promoter/coding sequence fusions as Pro35S:LFY. Use a single colon (:), not a double colon (::).Lowercase "p" should be used to refer to plasmids (e.g., pBR322), and to avoid confusion it should not be used to refer to promoters.

A double colon (::) should only be used for insertions (such as insertions by transposable elements), as in An1::dTph1, Bz1::Ac, or LFY::TAG1.

Transactivations should be written with >>, e.g., ProFIL>>.

Gene fusions corresponding to chimeric proteins should be indicated by a hyphen (e.g. Pro35S:PHT1;4-GFP)

**Genes and mutations.**

Among the most used nomenclature (including Arabidopsis, *Medicago truncatula*…), we find:

-WT gene symbol in three capital letters and written in italics *XXX1* (or full descriptive name with more letters)

-mutant gene symbol *xxx1-1,* with the last number specifying the mutant allele number 1 of gene *xxx1*

To avoid confusion, double or triple mutations should contain the names of the mutated genes separated by a space, e.g., *sad1 sad2*, or *cad4 cad5 cad6*, and should not be given entirely new names.”

PLANT AND SEED GENERATIONS

For **transgenesis:** the original plant used for the transformation process corresponds to the **T0** generation.

Seeds will be collected and sown to identify the first transformants. These plants correspond to the **T1** generation.

Subsequent generations (**T2, T3**, etc) will be obtained by successive **self-crossing**.

For chemical / physical **mutagenesis**, we must distinguish EMS from gamma mutations

For **EMS mutations**: seeds subjected to mutagenesis correspond to the **M1** generation.

Subsequent generations (**M2, M3**, etc) will be obtained by successive **self-crossing**.

**For CRISPR mutagenesis**, you might need to enter two independent mutations: seeds might contain a T-DNA insertion (containing the CRISPR cassette), which will cause the secondary mutations in the CRISPR locus. Generations are labelled following the initial T-DNA transformation (**using T1, T2, T3, etc, as with a regular transgenesis**), even if the initial T-DNA insert has subsequently been lost through selection.

For **gamma ray mutagenesis of pollen**: the mutagenesis is applied directly onto pollen (*i.e.*, the male parent), and this pollen will be used immediately to perform a crossing with a female parent. Since the first generation results from a crossing, it must be named **F1**. You must specify which parent is the female / male, by **always placing the reference to the female parent first**.

For example: F1 *mut5* X *drf3*.

Subsequent generations (**F2, F3**, etc) will be obtained by successive **self-crossing**.

If seeds (rather than pollen) are irradiated by gamma rays, follow the same rules as for EMS mutagenesis.

If you received seeds from a laboratory at an unknown generation stage, please use the letter x for this generation (for instance Tx, Fx, Mx). Successive generations will be Tx+1, Tx+2, or Mx+1, Mx+2, etc.

Any **crossing between two lines** results in the generation of an **F1**. You must specify which parent is the female / male, by **always placing the reference to the female parent first**.

For example: F1 *pht1;1* X *pht1;2*.

Subsequent generations (**F2, F3**, etc) will be obtained by successive **self-crossing**.

**Backcrosses** with a WT parent (same ecotype) are also crosses, and therefore result in an **F1**. The number of successive backcrosses should be specified.

For example: **B.C.X1 F1** (one back-cross), **B.C.X2 F1** (two back-crosses), etc.

Subsequent generations (**B.C.X1 F2, B.C.X1 F3**, etc) will be obtained by successive **self-crossing**.

Since these backcrosses should not affect the line properties (although they may!), you may simply enter corresponding plants and seeds in the original line table.

A **crossing between your mutant and WT plants from a distinct ecotype** requires the generation of a **new line**, which will reflect the mix between the two ecotypes.

UPLOADING A FILE

For **sequences**: use **.gb** (genbank) or **fasta** file format.

For **phenotypes**: use **word, powerpoint or tiff** file format.

General lab seed stock

You can keep most of your seed batches in your own boxes, but please place AT LEAST ONE ALIQUOT of your lines in the general lab seed stock.

To do this, simply place some seeds in a tube (NOT STERILIZED SEEDS, since sterilized seeds do not last very long). Go to the lab seed stock and identify a box with available space (if none are available, prepare a new box).

In the SEEDUSOON software, create a new seed batch for your line. **A new ID number will be automatically generated by the software** (see below). **Write this ID number on the top of the tube**.

**On the side of the tube, write the line name**.

Place the tube in the lab seed stock box, and write “**Lab Stock box N…”** in the area corresponding to the storage place in the SeedUSoon software.


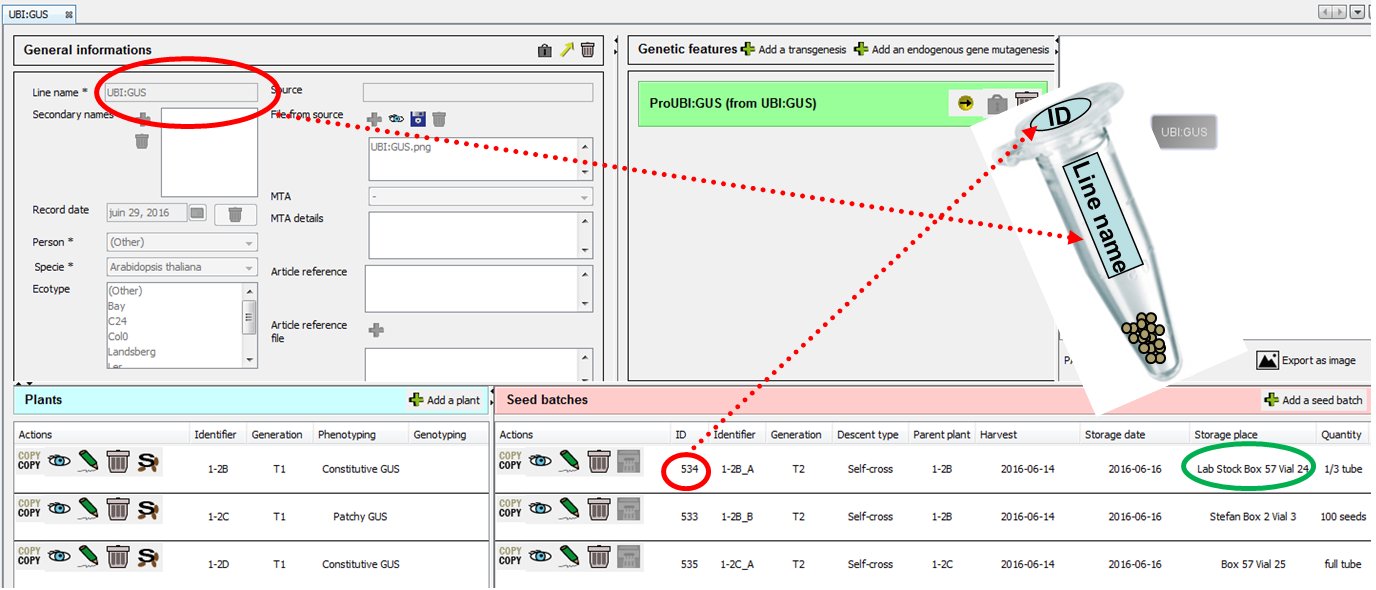


Since only a subset of all seed batches recorded in SEEDUSOON will be in the “General Lab Seed Stock”, gaps between ID numbers are absolutely normal.

SEED STORAGE CONDITIONS

How long your seeds will be able to germinate depends on the **seed storage conditions**; however it is also strongly affected by the seed genotype along with the **plant culture conditions**. For this reason: take care of your plants and seeds!

In particular, make sure to **limit the humidity while seeds are maturing**, and **allow seeds to dry in a well ventilated environment for 2-3 weeks** after harvest (avoid keeping your samples in non-breathing bags or tubes).

Depending on how long you intend to keep your seeds, you can:

-keep them at **room temperature** (**short term conservation** = a few months)

-keep them in the **seed storage room** with controlled humidity and temperature (**medium term conservation** = 1-3 years). Please add one aliquot of your lines to the “Stock commun LBDP”!

-**freeze them at -20C** in a screw-cap tube (**long term** conservation = more than 2 years)

Beware: **once sterilized, seeds lose their capacity to germinate within a few months**.

PLANT SELECTION

For Kanamycin, PPT and hygromycin selection, follow the protocol as in:

“A rapid and robust method of identifying transformed Arabidopsis

thaliana seedlings following floral dip transformation”

Harrison et al., Plant Methods 2006, 2:19 doi:10.1186/1746-4811-2-19

In addition, PPT resistant plants can be selected by spraying BASTA on young plantlets in soil. Follow the protocol as in: “Arabidopsis thaliana, a laboratory manual”

USEFUL LINKS

To learn about the function of an Arabidopsis gene, you can refer to:

<http://www.arabidopsis.org/servlets/Search?action=new_search&type=gene>

To learn about its expression pattern, refer to:

<http://bar.utoronto.ca/efp/cgi-bin/efpWeb.cgi>

To find citations related to your line or gene of interest, refer to:

<http://www.ncbi.nlm.nih.gov/pubmed/>

ORGANISMS CLASS

**Classification follows the European Community definition.** This classification separates infectious biological agents by risk group. (Source: Directive 2000/54EC of 18 September 2000 (O.J. L 262, 17.10.2000, p.21)

**Group 1:** unlikely to cause human disease.

**Group 2:** can cause human disease and might be a hazard to workers; it is unlikely to spread to the community; there is usually effective prophylaxis or treatment available.

**Group 3:** can cause severe human disease and present a serious hazard to workers; it may present a risk of spreading to the community, but there is usually effective prophylaxis or treatment available.

**Group 4:** causes severe human disease and is a serious hazard to workers; it may present a high risk of spreading to the community; there is usually no effective prophylaxis or treatment available.

A problem with the software (bug, suggestion)?

Contact the development team (GIPSE) at:

[SeedUSoon@cea.fr](mailto:kiptrak@cea.fr)
