# Supplementary material for: SeedUSoon: A New Software Program to Improve Seed Stock Management and Plant Line Exchanges between Research Laboratories
Source: Front Plant Sci. 2017 Jan 20;8:13. doi: 10.3389/fpls.2017.00013 (PMC5247430; doi:10.3389/fpls.2017.00013)
Supplement: Supplementary file 2 [file Image_1.PDF]

Legend	
Required	R
Optional	*
Exported	↗
Deleted during export	✗

Field			TRANSGENESIS	ENDOGENOUS GENE MUTAG.
GENERAL INFORMATION FIELDS				
Line name (and secondary names)	R ↗			
Record date	* ✗			
Person	R ✗			
Species	R ↗			
Ecotype	* ↗			
Source	* ↗			
File from Source	* ↗			
MTA	* ↗			
MTA details	* ✗			
Article reference (without attached files)	* ↗			
GENETIC FEATURE FIELDS				
Designation			R ↗	R ↗
Short description			* ↗	* ↗
Gene name (and secondary names)			* ↗	* ↗
Method			* ↗	* ↗
Method reference			* ↗	* ↗
Transgene sequence (with attached file)			* ↗	
Organism class			* ↗	
Pathogenicity			* ↗	
Selectable marker in plants			* ↗	
Strain			* ↗	* ↗
Dose				* ↗
Mutation sequence				* ↗
Sequencing realized by (+ Notebook ref)			* ✗	* ✗
Genotyping protocol (with attached file)			* ↗	* ↗
Genotyping realized by (+ Notebook ref)			* ✗	* ✗
PLANTS (P) AND SEED BATCHES (S) FIELDS				
ID (automatically generated)		*S*	R ✗	R ✗
Identifier		*P/S*	R ↗	R ↗
Generation		*P/S*	* ↗	* ↗
Descent type (backcross/selfcross)		*S*	* ↗	* ↗
Parent plant		*S*	* ↗	* ↗
Seed batches collected from a particular plant		*P*	* ↗	* ↗
Phenotyping result (with attached file)		*P/S*	* ↗	* ↗
Phenotyping realized by (+ Notebook ref)		*P/S*	* ✗	* ✗
Genotyping		*P*	* ↗	* ↗
Genotyping realized by (+ Notebook ref)		*P*	* ✗	* ✗
Insertion loci sequences (with attached files)		*P*	* ↗	
Insertion sequencing realized by (+ Notebook ref)		*P*	* ✗	
Harvest date		*S*	* ↗	* ↗
Storage date, storage place and quantity		*S*	* ✗	* ✗
Harvesting realized by (+ Notebook ref)		*S*	* ✗	* ✗
Germination assay		*S*	* ↗	* ↗
Germination assay realized by (+ Notebook ref)		*S*	* ✗	* ✗
Genetic feature segregation profile		*S*	* ↗	* ↗
Segregation profile realized by (+ Notebook ref)		*S*	* ✗	* ✗
Comments		*P/S*	* ↗	* ↗

SupFig. 1: **Fields available in the user mode for the "General information" area, individual "Genetic features", and plant or seed batches entries.** Required or optional entries are indicated, along with a specification of the data conserved during plant line export.
